# Screening Eating Disorders Among Female High School Students in Makkah City: A Cross-Sectional Survey

**DOI:** 10.7759/cureus.34888

**Published:** 2023-02-12

**Authors:** Nasser Al Shanbari, Abdulrahman Alharthi, Salah Bakry, Safaa Alsalmi, Raghad Saleh, Gufran Kambiji, Jomanah Saleh, Ethar Alsulami, Mokhtar Shatla

**Affiliations:** 1 Department of Medicine and Surgery, College of Medicine, Umm Al-Qura University, Makkah, SAU; 2 Department of Family and Community Medicine, College of Medicine, Umm Al-Qura University, Makkah, SAU

**Keywords:** psychiatric disorder, prevalence, medicine, female, bulimia nervosa, anorexia nervosa

## Abstract

Introduction: Eating disorders (EDs) are defined as disturbances in health and psychosocial functioning. They are more predominant in young and adult women and can start at an early age and continue into adulthood. These types of mental illnesses can be prevented with proper training. However, there is a significant lack of understanding of EDs among teachers. Therefore, this study aimed to estimate the prevalence rates of EDs among female high school students in Makkah city.

Methods: We surveyed 384 female students from high schools in Makkah city in this cross-sectional study using a validated questionnaire adapted from a previously published study after translating it into Arabic.

Results: Most of the students had suffered an ED (n=211, 54.4%), while 45.6% had not. There was an insignificant relation between the frequency of EDs and participants’ age, nationality, family history of ED, and awareness of EDs (P-value, 0.158, 0.486, 0.671, and 0.718, respectively).

Conclusion: Further studies are needed to evaluate the understanding of EDs among the general population as well as assess the prevalence rates of EDs among larger female populations.

## Introduction

Eating disorders (EDs) are psychiatric disorders defined as abnormal weight control and eating habits, which are also characterized by disturbances in health and psychosocial functioning [1,2]. EDs affect men and women during adolescence and early adulthood [2]. However, rates are higher in girls and adult women aged 18-24 years [2,3]. Indeed, EDs can start as early as six years and continue into adulthood [2,3].

In western countries, the prevalence rates of anorexia nervosa and bulimia nervosa among young women are 0.3% and 1%, respectively [2]. Studies in the Kingdom of Saudi Arabia and other Arab countries [2,4-6] as well as internationally [2,7] report that up to 24% of young women suffer mental health problems such as anxiety, depression, and behavioral disorders [2,8,9].

The early detection of EDs in school-aged children is essential since teachers, with the proper training, can significantly help prevent such illnesses [10]. However, when implementing ED prevention programs in schools, teachers typically show a significant lack of understanding since they receive little training on healthy eating habits and ED prevention approaches [10,11]. This could contribute to increasing the prevalence rates of EDs further. As young adult women have higher prevalence rates of EDs outside Saudi Arabia, in this study, we aimed to estimate the prevalence rates of ED among female high school students in Makkah city, Saudi Arabia.

## Materials and methods

Study design and ethical consideration

We surveyed female high school students in Makkah city from September 20 to October 15, 2022 after being granted ethical approval by UQU’s research ethics committee in September 2022 (approval number HAPO-02-K-012-2022-09-1178). Study setting and population: After collecting the names of high schools in the Makkah region from the website of the Ministry of Education, we divided them into seven categories by region: the north, south, east, west, central, Bahrah, and Al-Jamome regions. Then, we used the website Random.org to select two schools from each region randomly. After this stratification, we included only female students from the chosen high schools in Makkah city and excluded those who refused to participate in the survey.

Sample size

The sample size was calculated using Epi Info™ 7.1.5 (Centers for Disease Control and Prevention, Atlanta, Georgia); therefore, the sample size of female high school students was 388 with a confidence interval of 95% [12].

Data collection tool

The study tool used to investigate EDs in this study was adapted from a validated questionnaire adopted in a previous study; this was translated into Arabic by a bilingual translator followed by backward translation [13]. A Google platform (the newest version provided) was used to distribute the survey online using the WhatsApp groups of the students after contacting the schools’ administration for permission. Online written informed consent for participation in the survey was received from all the participants. The WhatsApp number of the corresponding author was provided with the survey for any inquiries.

Data analysis

We first entered our data into Microsoft Excel spreadsheets to ensure their completeness and check for minor typographical mistakes. Then, we uploaded data into a Statistical Package for Social Studies v.23 spreadsheets (IBM, Armonk, NY). Descriptive statistics were defined as percentages for the categorical data and the mean and standard deviation for the continuous data. A significance level of <0.05 was considered to be statistically significant. The Chi-square test was used to compare the categorical variables.

## Results

Sociodemographic analysis

Female high school students in Makkah answered this screening survey. The mean age of the 388 students was 17.5 years (SD=5.23). Most of the students were aged 18 years or less (n=363, 93.6%), while Saudi participants provided more responses (n=334, 86.1%) than non-Saudi participants (Table 1). Most of the students had no history of EDs (n=351, 90.5%), with only 9.5% having previously suffered an ED. Moreover, most had a negative family history of EDs (n=366, 94.3%), while a minority had a family history of EDs (5.7%). Furthermore, most of the students had previously heard about EDs, representing a good awareness (n=271, 69.8%), while only 30.2% had an inadequate awareness (Table 1).

**Table 1:** Demographic data on the respondents (n=388) Age = (Mean=17.5), (SD=5.23)

Categories		N.	%
Age groups	18 years or less	363	93.6
	More than 18	25	6.4
Nationality	Saudi	334	86.1
	Non-Saudi	54	13.9
Past history of ED	Yes	37	9.5
	No	351	90.5
Family history of ED	Yes	22	5.7
	No	366	94.3
Awareness of ED	Yes	271	69.8
	No	117	30.2

Descriptive analysis

This survey screened female students with EDs using the SCOFF questionnaire. In the SCOFF questionnaire, we classified the questions as items 1-5. Two additional items were used to assess bulimia nervosa symptoms, which were classified as items 6 and 7. These items are shown in Table 2. Items 1, 3, 4, and 5 showed a negative response at proportions of 81.2%, 70.1%, 58%, and 60.6%, respectively, while item 2 showed a positive response at a proportion of 51.8%. Furthermore, the bulimia nervosa items (items 6 and 7) showed predominantly negative responses (52.6% and 83.2%, respectively) (Table 2). Over half of the students had an ED (n=211, 54.4%), while 45.6% did not (Table 2).

**Table 2:** Frequency of ED items to investigate the prevalence rates of EDs among female students

Categories		N.	%
Do you make yourself Sick because you feel uncomfortably full?	No	315	81.2
	Yes	73	18.8
Do you worry you have lost Control over how much you eat?	No	187	48.2
	Yes	201	51.8
Have you recently lost more than One stone (6.35 kg) in a three-month period?	No	272	70.1
	Yes	116	29.9
Do you believe yourself to be Fat when others say you are too thin?	No	225	58.0
	Yes	163	42.0
Would you say Food dominates your life?	No	235	60.6
	Yes	153	39.4
Are you satisfied with your eating patterns?	No	204	52.6
	Yes	184	47.4
Do you ever eat in secret?	No	323	83.2
	Yes	65	16.8
The likelihood ratio of ED screening among participants	Positive	211	54.38
	Negative	177	45.62

Inferential statistical test

Table 3 shows the association between the participants’ demographics and their frequency of EDs. Those participants with and without a history of EDs corresponded significantly with a positive likelihood ratio of an ED (n=29 and n=182, respectively) (P-value, 0.002). On the other hand, participants’ age, nationality, family history of ED, and awareness of EDs showed insignificant relations with the frequency of EDs (p-value, 0.158, 0.486, 0.671, and 0.718, respectively) (Table 3).

**Table 3:** Association between the participants’ demographic characteristics and their likelihood of having an ED

Categories		Screening of ED				P-value
		Negative		Positive		
Age groups	18 years or less	169		194		0.158
	More than 18	8		17		
Nationality	Saudi	150		184		0.486
	Non-Saudi	27		27		
Past history of ED	Yes		8		29	0.002*
	No		169		182	
Family history of ED	Yes		11		11	0.671
	No		166		200	
Awareness of ED	Yes		122		149	0.718
	No		55		62	

## Discussion

Our study aimed to estimate the prevalence rates of EDs among female high school students in Makkah city, Saudi Arabia. The study’s results showed that 54.4% of the students screened had previously experienced ED symptoms. Another study in Dammam showed that 29.4% of female students were classified as having a high level of concern for an ED [1]. Furthermore, a review paper stated that most recent studies have confirmed the high prevalence rates of EDs, especially among women [14]. The same study also confirmed the increased prevalence rates of EDs in Asia and developing Middle Eastern countries [14]. Another previous study assessed eating attitudes and body image concerns among women, finding that 24% of them scored as having a possible ED and 74.8% were dissatisfied with their body image [4].

According to our study’s findings, most of those respondents who had suffered an ED were 18 years old or less; Saudis were also more predominant among this group. The findings of a recent review article reported that the incidence rate has increased among people aged under 15 years [15]. Therefore, we recommend further studies assessing the prevalence rates of EDs and their association with numerous factors such as age and sex to enhance the early detection of these disorders.

Surprisingly, most of the respondents who had suffered an ED denied having a previous history of EDs. However, many confirmed their awareness of EDs, indicating a questionable level of awareness among the study population. This finding correlates with that of another study that showed a low level of knowledge of EDs, with 59.4% and 39.8% of its participants failing to recognize the correct definition of anorexia and bulimia nervosa, respectively [16]. Hence, further studies assessing the understanding of EDs among the general population are recommended in addition to programs and campaigns to raise awareness of EDs. Recent studies have confirmed the increased mortality risk and burden of EDs since they disable 3.3 million people worldwide annually [15,17]. Hence, screening measures and early prevention approaches are highly suggested.

Strengths and limitations

In our study, we used a precise and short tool to assess the prevalence rates of EDs among our population. However, applying this tool to a larger population would result in more valuable findings. Additionally, recall bias was possible since this was a cross-sectional study based on an online questionnaire.

## Conclusions

The findings of our study confirm the concerning prevalence rates of EDs among female students, accounting for more than half of the study population. Moreover, there was a questionable level of awareness of EDs, which indicates the need for further studies evaluating the understanding of EDs among the general population. Given the high prevalence rates in our study, we recommend additional studies assessing the prevalence rates of EDs among larger female populations.
